# Laser Feedback Interferometry as a Tool for Analysis of Granular Materials at Terahertz Frequencies: Towards Imaging and Identification of Plastic Explosives

**DOI:** 10.3390/s16030352

**Published:** 2016-03-09

**Authors:** She Han, Karl Bertling, Paul Dean, James Keeley, Andrew D. Burnett, Yah Leng Lim, Suraj P. Khanna, Alexander Valavanis, Edmund H. Linfield, A. Giles Davies, Dragan Indjin, Thomas Taimre, Aleksandar D. Rakić

**Affiliations:** 1 School of Information Technology and Electrical Engineering, The University of Queensland, Brisbane QLD 4072, Australia; s.han@uq.edu.au (S.H.); bertling@itee.uq.edu.au (K.B.); ylim@itee.uq.edu.au (Y.L.L.); 2 School of Electronic and Electrical Engineering, University of Leeds, Leeds LS2 9JT, UK; P.Dean@leeds.ac.uk (P.D.); el08jk@leeds.ac.uk (J.K.); A.D.Burnett@leeds.ac.uk (A.D.B.); khannasp@nplindia.org (S.P.K.); A.Valavanis@leeds.ac.uk (A.V.); E.H.Linfield@leeds.ac.uk (E.H.L.); g.davies@leeds.ac.uk (A.G.D.); D.Indjin@leeds.ac.uk (D.I.); 3 School of Chemistry, University of Leeds, Leeds LS2 9JT, UK; 4 School of Mathematics and Physics, The University of Queensland, Brisbane QLD 4072, Australia; t.taimre@uq.edu.au

**Keywords:** terahertz imaging, interferometry, semiconductor lasers, quantum cascade, optical constants

## Abstract

We propose a self-consistent method for the analysis of granular materials at terahertz (THz) frequencies using a quantum cascade laser. The method is designed for signals acquired from a laser feedback interferometer, and applied to non-contact reflection-mode sensing. Our technique is demonstrated using three plastic explosives, achieving good agreement with reference measurements obtained by THz time-domain spectroscopy in transmission geometry. The technique described in this study is readily scalable: replacing a single laser with a small laser array, with individual lasers operating at different frequencies will enable unambiguous identification of select materials. This paves the way towards non-contact, reflection-mode analysis and identification of granular materials at THz frequencies using quantum cascade lasers.

## 1. Introduction

The rapid, automatic, non-contact detection of explosives in non-cooperative security scenarios remains a challenge with significant practical benefit. Coherent sensing in the terahertz (THz) frequency range shows strong potential as a means to overcome this challenge, in particular due to its non-ionising nature and the unique spectral features of many explosives in this frequency band [1,2,3,4]. Laser feedback interferometry (LFI), championed by Donati and co-workers over the past four decades [5,6], provides a simple coherent sensing methodology which permits non-contact interrogation of remote targets [7]. The use of LFI at THz frequencies offers an ultimately compact platform for coherent THz sensing, without the need for an external detector [8,9,10].

We previously demonstrated a method for materials analysis at THz frequencies using LFI with a THz quantum cascade laser (QCL) [11,12]. We successfully applied this technique to homogeneous organic materials, relying on the spatial homogeneity and simple ensemble averages to reduce natural variability of optical constants over scanned areas of homogeneous materials, thereby enabling their successful recovery. A particularly useful visual representation of the optical characteristics of materials under test is the (two-dimensional) distribution of the magnitude and phase of their reflection coefficients measured at different locations on the material surface. When observed, these distributions show little deviation in either magnitude or phase and can be represented by distributions concentrated tightly around their respective centroids.

However, there is a large class of systems which are granular in nature, including plastic explosives, where the internal dielectric heterogeneity creates electromagnetic response very different from those of its constituent materials [13]. When the size of constituent grains in a granular system is on the order of the wavelength of the incident electromagnetic wave, the effective optical properties cannot be extracted using the algorithms developed for homogeneous materials; problems associated with extracting effective optical properties of such materials are well-recognised [13,14,15,16]. Plastic explosives are comprised of an explosive compound or a mixture of explosives combined with a variety of plasticizers, desensitizers, dyes, waterproof coatings, and fabrics to aid storage and use [17]. These materials, frequently referred to as random granular systems, can be modelled as a mixture of explosive crystal grains and air voids embedded in an inert matrix [13,18]. In such systems, both the air voids and explosive crystals are significant sources of dielectric heterogeneities.

We deal with the random nature of grains in the system by interrogating the sample at a number of spatially distinct points, therefore acquiring a number of signals containing local information at those points. The random nature of the system creates a set of measurements likely to contain outliers, necessitating the use of a robust estimator for the extraction of the effective optical constants of the material. Indeed, this is the case for the plastic explosives used in this study. In this article, we develop a self-consistent algorithm to extract the effective optical properties of random granular systems; the process requires the removal of phase uncertainties, reliable location of centroids (representative of the effective optical constants), and is executed in an unsupervised, fast, and robust manner. We then demonstrate the effectiveness of this approach by successfully extracting optical constants of three plastic explosives. We further show that performs equally well on three homogeneous plastics.

The remainder of this article is structured as follows: In Section 2 we detail the experimental set-up, and explain our approach and present our results in Section 3 and Section 4. We draw conclusions in Section 5.

## 2. Experimental Setup and Procedure

In LFI, a portion of the emitted beam is coupled back into the laser cavity after reflection from an external target. This optical feedback affects the laser’s operating parameters; in particular, the laser emission frequency and the voltage across the laser terminals. With a fixed external target, modulating the laser bias current induces a modulation of the laser emission frequency. The laser terminal voltage is then modulated in two ways: (1) directly by the modulating current; and (2) indirectly by the optical feedback.

Our technique exploits the way in which the complex refractive index of the remote target affects this indirect modulation of the laser terminal voltage due to optical feedback. We refer to this interferometric voltage waveform (temporal variation of laser voltage) as the self-mixing (SM) signal. When the slow laser bias current sweep induces a linear frequency sweep, the relationship between SM signal and complex index is particularly simple. This permits the recovery of the complex refractive index—$\hat{n}=n-j\;k$ where *n* is the refractive index and *k* is the extinction coefficient—of an unknown material sample embedded in an optically flat target aligned perpendicular to the optical axis, using the known complex refractive indices of two other material samples embedded in the same target.

To demonstrate our technique, we prepared a custom target with three plastic explosive samples embedded in a polymer holder similar to our previous work [11]. The samples were gently compressed against an optically flat reference plane (removed during measurements) in order to minimize tilt and position effects [12] creating a flat 3 mm diameter surface for scanning. All of the three samples were granular systems: SX2 [1,3,5-trinitroperhydro-1,3,5-triazine (RDX) based], Metabel [1,3-dinitrato-2,2-bis(nitratomethyl)propane (PETN) based], and Semtex-H (RDX and PETN based).

Material from each of the plastic explosives from the same batch was separately made into pellets and measured using THz time domain spectroscopy (THz-TDS) (in transmission geometry) at nine points (in a 3×3, 0.5 mm grid) across each sample. Average values at 2.62 THz were extracted and used as the reference values for the LFI measurements.

**Figure 1:**
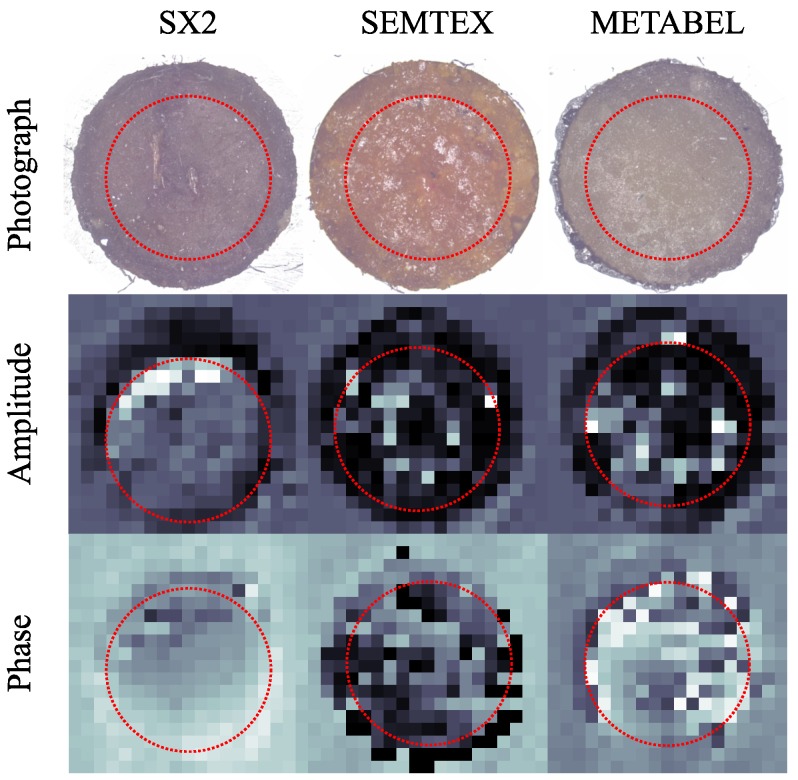
Plastic explosive samples. First row: microscope images; Second row: terahertz (THz) amplitude-like images; Third row: THz phase-like images.

The THz QCL device used in our experiments was a 10-µm-thick GaAs–AlGaAs bound-to-continuum active region, [19] processed into a semi-insulating surface-plasmon ridge waveguide with dimensions 3 mm × 140 µm, operating at 2.6 THz, at a heat sink temperature of 25 K. The custom target was aligned perpendicular to the optical axis at a distance 410 mm from the laser, and scanned in a raster fashion. At each point of the scan, the laser bias current was linearly modulated between 930 mA and 1130 mA in a saw-tooth pattern, resulting in an almost linear frequency change with time. The frequency of the saw-tooth modulation waveform was 1 kHz, and the frequency modulation coefficient of the laser used was 15 MHz/mA. At each point in the raster scan the current sweep was repeated 64 times and an average voltage waveform was recorded. Repeating this process at each point results in a two-dimensional array of interferometric voltage signals. The removal of the common voltage slope—“negatisation” [20] results in an array of SM signals [11]. This two-dimensional array of SM signals was then processed to create amplitude-like and phase-like images for the plastic explosive samples (see Figure 1).

By inspection of the SM signal array, we observe that all signals have been acquired in the weak feedback regime (C≤1), which is a characteristic frequently associated with QCLs [21]. For further processing, this array of SM signals is smoothed by using a local moving average algorithm.

## 3. Processing the Self-Mixing Signals

Through a series of considered steps, the SM signals for each granular system can be processed to obtain a single effective complex refractive index proxy (an amplitude-like/phase-like pair which contains the complex reflectivity information).

We begin by recalling the excess phase equation, which relates the round trip phase in the external cavity at the frequency of the unperturbed laser $\varphi_{S}$ to the phase of the laser with feedback $\varphi_{\mathrm{FB}}$:

(1) $$\varphi_{S}-\varphi_{\mathrm{FB}}=C\;\sin\left(\varphi_{\mathrm{FB}}+\arctan\alpha \right)\;$$

where *C* is the feedback parameter and *α* is the linewidth enhancement factor [22]. The SM voltage signal *V* can be modelled as [11]

(2) $$V=V_{0}+\beta \cos\left(\varphi_{\mathrm{FB}}\right)\;$$

where $V_{0}$ is a voltage offset which may differ from point to point on the target, *β* is the modulation coefficient of the SM signal, and $\varphi_{\mathrm{FB}}$ satisfies Equation (1).

In our framework, the round trip phase in the external cavity $\varphi_{S}$ has three components:

(3) $$\varphi_{S}\left(t\right)=\theta_{0}+\frac{\Phi }{T}\;t-\theta_{R}\;$$

where $\theta_{0}$ is the constant phase shift on transmission accumulated at the start of the frequency sweep, Φ is the phase modulation coefficient for the current sweep, and $\theta_{R}$ is the phase shift on reflection from the target.

We can now rewrite Equation (1) as:

(4) $$\theta_{0}+\frac{\Phi }{T}\;t-\theta_{R}-\varphi_{\mathrm{FB}}=C\;\sin\left(\varphi_{\mathrm{FB}}+\arctan\alpha \right)\;$$

or by expanding the trigonometric term as:

(5) $$\theta_{0}+\frac{\Phi }{T}\;t-\theta_{R}-\varphi_{\mathrm{FB}}=\frac{C}{\sqrt{1+\alpha^{2}}}\;\sin\left(\varphi_{\mathrm{FB}}\right)+\frac{C\alpha }{\sqrt{1+\alpha^{2}}}\;\cos\left(\varphi_{\mathrm{FB}}\right)\;$$

This may be rewritten using vector notation as:

(6) $$\frac{\Phi }{T}\;t-\varphi_{\mathrm{FB}}=\left[\begin{array}{ccc} \theta_{R}-\theta_{0}, & \frac{C}{\sqrt{1+\alpha^{2}}}, & \frac{C\alpha }{\sqrt{1+\alpha^{2}}} \end{array}\right]\left[\begin{array}{c} 1\\ \sin\left(\varphi_{\mathrm{FB}}\right)\\ \cos\left(\varphi_{\mathrm{FB}}\right) \end{array}\right]\;$$

We assume that *C* and *α* may be treated as constant throughout the (small) frequency sweep. Therefore, if Φ/T and $\varphi_{\mathrm{FB}}$ are known, then Equation (6) is a linear equation in $\left[\theta_{R}-\theta_{0},C/\left(\sqrt{1+\alpha^{2}}\right),C\alpha /\left(\sqrt{1+\alpha^{2}}\right)\right]$ for *every* time point *t*. It is then straightforward to obtain these coefficients from this over-determined system of equations using the method of least squares. The pair $\left[C/\left(\sqrt{1+\alpha^{2}}\right),\theta_{R}-\theta_{0}\right]$ acts as a proxy for the complex refractive index $\left[n,k\right]$ of the target associated with the SM signal [11,12].

**Figure 2:**
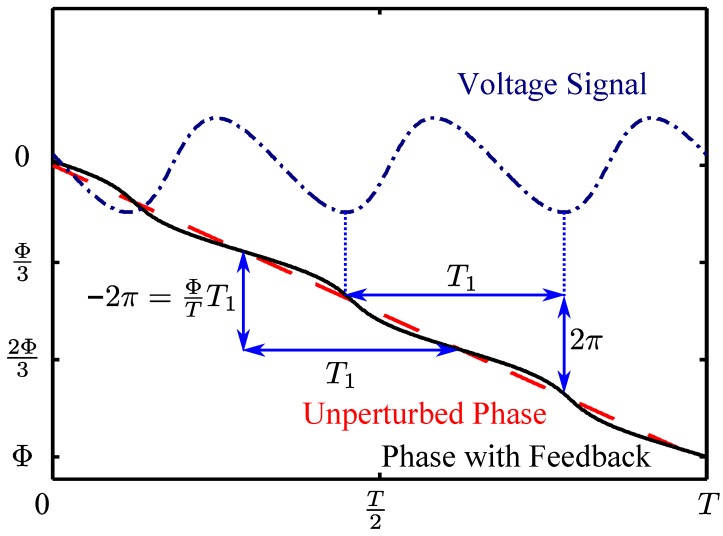
The blue chain line is the waveform of the self-mixing voltage signal, the black solid line is the phase of the laser with feedback, the red broken line is the the round trip phase delay in the external cavity.

The value of Φ/T can be obtained directly from the SM voltage signal, as we now explain. Suppose the period of the SM voltage signal is $T_{1}$, as shown in Figure 2. From Equation (2) we see immediately that, for each period $T_{1}$ of the voltage signal, the phase $\varphi_{\mathrm{FB}}$ changes by exactly 2π. When C≤1, Equation (1) has only one solution [23]. Consequently, a change in $\varphi_{\mathrm{FB}}$ of 2π over a period $T_{1}$ necessitates the same change in $\varphi_{S}$ in Equation (1), which we see from Equation (3) implies that $\Phi T_{1}/T=2\pi$, from which Φ/T can be obtained. Note that it is straightforward to extract the period of the SM voltage signal $T_{1}$ by taking the overall average delay between successive peaks and successive troughs of the SM signals.

The phase under feedback $\varphi_{\mathrm{FB}}$ was recovered from the voltage signal modelled by Equation (2). For each half-period of the SM signal—corresponding to alternating rising and falling portions (see Figure 2)—we can approximately invert Equation (2) through:

(7) $$\varphi_{\mathrm{FB}}\left(t\right)=\pm \arccos\left[\frac{V\left(t\right)-\left(\frac{V_{\max}+V_{\min}}{2}\right)}{\left(\frac{V_{\max}-V_{\min}}{2}\right)}\right]+2\pi n\;$$

where $V_{\min}$ and $V_{\max}$ are the minimum and maximum of the half-period of the SM voltage signal under consideration, respectively, and the integer *n* (initially zero) captures which fringe the signal is associated with at time *t*. The positive solution of Equation (7) corresponds to a rising portion of *V* and the negative solution corresponds to a falling portion of *V*.

## 4. Extraction of Optical Constants of Materials

For each granular system, a representative set of SM signals was selected according to the following three steps. Firstly, only SM signals which purely contain information pertaining to the optical properties of each granular system were selected (in this case within a radius of six pixels from the center of each material, that is those contained within the dotted circles in Figure 1). This process ensures that selected SM signals were not affected by optical properties of the sample holder, nor the interface between the sample and the holder. Secondly, the parameter extraction was performed according to the procedure outlined above for each of the selected SM signals. Only highly representative fits were retained—fits corresponding to the largest 5% of the residual errors between the SM signal and the fitted curve for each of the samples were discarded. Thirdly, the signal fits with the largest 1% of the extracted feedback parameter *C* (suggesting unusually high reflectivity associated with the signal) were also discarded.

At the conclusion of these three steps, we have obtained an array of fitted parameters *C*, *α*, and $\theta_{R}-\theta_{0}$ for the retained pixels of each material sample. This set of parameters has a linear relationship with the reflectivity and phase-shift on reflection of each material.

However, the problem is compounded by the phase values extracted from Equation (6). These phase values must be concentrated within one 2π period for the algorithm to be effective. Indeed, this was the case for all materials explored here.

**Figure 3:**
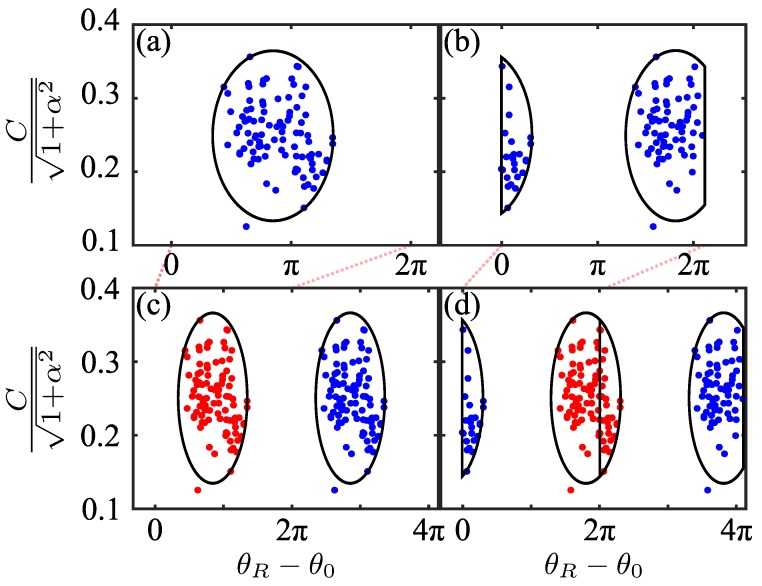
The point cloud corresponding to $\left[C/\left(\sqrt{1+\alpha^{2}}\right),\theta_{R}-\theta_{0}\right]$ pairs for SX2. (**a**) the point cloud does not experience phase wrapping (ideal case); (**b**) the point cloud experiences phase wrapping as it extends beyond 2π (more typical result); (**c**,**d**) show the point clouds of (**a**,**b**) together with a copy shifted by 2π.

The two possible scenarios for the extracted phase distribution that can arise, yet still be resolved, are visualised in Figure 3. Figure 3a,b depict these possible point clouds of $\left[C/\left(\sqrt{1+\alpha^{2}}\right),\theta_{R}-\theta_{0}\right]$ pairs. To automatically resolve this ambiguity for each material, we consider $\theta_{R}-\theta_{0}$ modulo 2π (${\tilde{\theta }}_{R}=\theta_{R}-\theta_{0}$), and then duplicate the array $\left[C/\left(\sqrt{1+\alpha^{2}}\right),{\tilde{\theta }}_{R}\right]$ phase-shifted by 2π—$\left[C/\left(\sqrt{1+\alpha^{2}}\right),{\tilde{\theta }}_{R}+2\pi \right]$. Considered together, the resulting array may contain either two clusters of points (the range of the fitted $\theta_{R}-\theta_{0}$ is within 2π), as in Figure 3c, or three clusters of points (the range of the fitted $\theta_{R}-\theta_{0}$ is not within 2π), as in Figure 3d.

In order to automatically determine which case arises in a particular situation (two clusters or three clusters), as well as which single representative point cloud to choose for each material (indicated as the red cloud in Figure 3c,d), we proceed as follows.

The K-Means algorithm [24,25] is a well known efficient procedure for obtaining the set number of clusters—two and three in these cases. To determine which of the clustering outcomes is the one we seek, we use the Silhouette Coefficient, which permits us to determine which clustering outcome gives correct division of the data [26]. We select either the left-most cluster (if it is determined that there are two clusters), or the centre cluster (if it is determined that there are three clusters) (for example the red points in Figure 3c,d).

**Figure 4:**
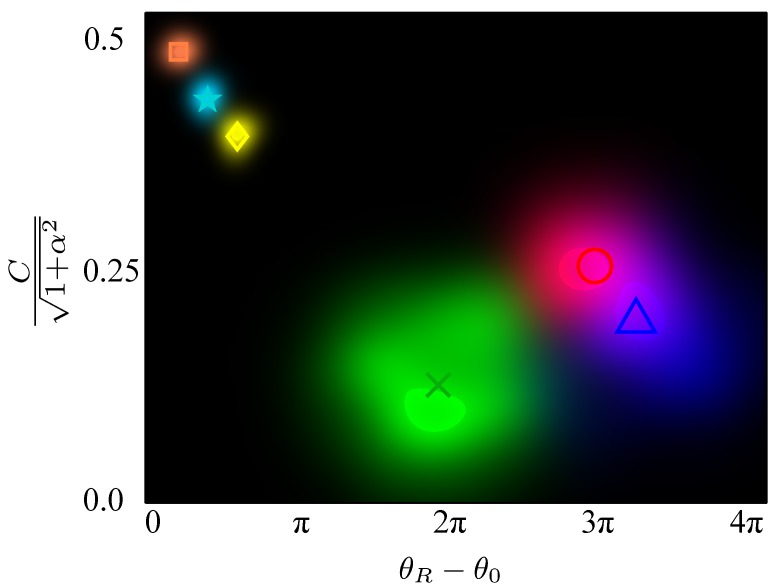
The distribution of the point cloud together with the centroid for the three plastics explosives: METABEL, SEMTEX, SX2 (indicated by red, green, blue clouds and circle, cross, triangle markers, respectively). Also shown for comparison are the point clouds for three homogenous plastics HDPE, PC, HDPE Black (indicated by orange, cyan, yellow clouds and square, star, diamond markers, respectively).

Now we have determined a unique set of points (between 80 and 100 for the plastic explosives in this study) with phase spread distribution well within 2π, and seek to extract a single pair of coordinates representative of the material under test. While the arithmetic average would be a natural choice for each coordinate in the pair, it is far too susceptible to outliers. Therefore, we opt for a measure of the centre of gravity of the point cloud obtained as follows. We apply the Mean-Shift algorithm iteratively [27,28] with a kernel chosen to be the reciprocal of the square of the Euclidean distance between the point pair and the previous iteration’s centroid (for the first iteration the arithmetic mean was used). Density plots for the selected cluster for each granular material, together with their centroid, are shown in Figure 4: METABEL, SEMTEX, SX2 (indicated by red, green, blue clouds and circle, cross, triangle markers, respectively). For comparison, we concurrently plot density plots for three homogeneous plastics: HDPE (High density polyethylene), PC (polycarbonate), HDPE Black (HDPE with black dye) (indicated by orange, cyan, yellow clouds and square, star, diamond markers, respectively). Note that the homogeneous plastics exhibit far less variability.

Finally we apply our materials analysis procedure to convert the automatically determined centroids into [n,k] pairs using the procedure described in [11,12], and verify the self-consistency of our scheme. The results of this procedure are presented in Table 1.

The aim of the study was to extract [n,k] values for granular materials; results shown in Table 2 demonstrate that the proposed algorithm is indeed effective for extracting *n* and *k* for the three plastic explosives in the study. The results for homogeneous plastics are presented mainly as an illustration. Referring to Figure 3 one can see that the distribution for these materials has considerably smaller variance than the ones for our carefully prepared samples containing plastic explosives. Nevertheless, the positions of the centroids of the representative clouds (notwithstanding their significantly larger variance) for plastic explosives and the [n,k] pairs extracted from them agree remarkably well with the values measured using the conventional TDS system.

We wish to point out again that the method described here is for materials analysis at a single frequency and enables the extraction of the corresponding [n,k] pair. In order to identify a particular material, more than one frequency would be required in order to capture its spectral signature. The array implementation of the scheme, with appropriately selected emission frequencies, would allow the identification or detection of specific compounds without ambiguity.

## 5. Conclusions

To conclude, we have introduced a self-consistent method for the analysis of granular materials at THz frequencies using a QCL. The method is designed for signals acquired from a laser feedback interferometer based on a THz QCL, and applied to non-contact reflection-mode sensing. Our technique was demonstrated using three plastic explosives samples, achieving good agreement with reference measurements obtained by transmission mode THz-TDS. This work lays the foundation for non-contact identification of granular materials at THz frequencies. This could be achieved by replacing a single laser with a small laser array, with individual lasers operating at different frequencies to enable unambiguous identification of select materials. Alternatively, one could employ a single laser with wide tuning range.

## Figures and Tables

**Table 1:** Literature reference values and LFI estimated values for *n* and *k* for the three homogeneous plastics. For source of literature values see [11].

	Lit. *n*	LFI *n*	Lit. *k*	LFI *k*
HDPE	1.54	1.54	0.002	0.006
PC	1.62	1.62	0.01	0.02
HDPE Black	1.58	1.58	0.02	0.02

**Table 2:** Values for *n* and *k* for the three plastic explosives: comparison of TDS and laser feedback interferometry (LFI) measurements.

	TDS *n*	LFI *n*	TDS *k*	LFI *k*
SX2	1.75	1.76	0.09	0.09
SEMTEX	1.55	1.56	0.06	0.07
METABEL	1.66	1.66	0.07	0.06
